# Supplementary figures and images for: SARS-CoV-2 replicates and displays oncolytic properties in clear cell and papillary renal cell carcinoma
Source: PLoS One. 2023 Jan 3;18(1):e0279578. doi: 10.1371/journal.pone.0279578 (PMC9810192; doi:10.1371/journal.pone.0279578)

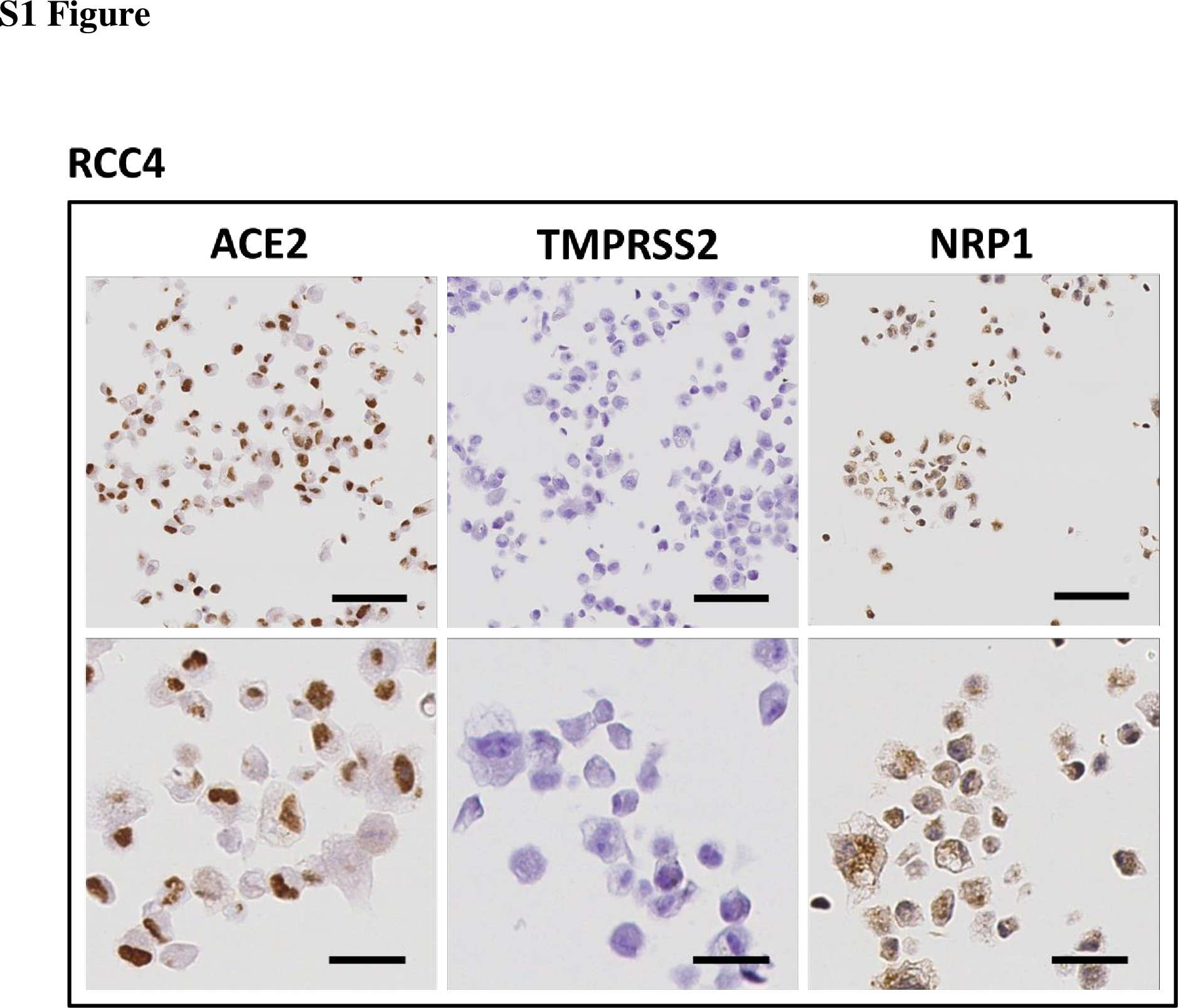

Supplement: S1 Fig — None of the proteins could be detected at the expected location in the CCRCC cell line RCC4. ACE2 displayed nuclear positivity, NRP1 a faint cytoplasmic staining, whereas TMPRSS2 was negative. Upper panel scale bar = 100μm, lower panel scale bar = 40μm. (TIF) [file pone.0279578.s001.tif]

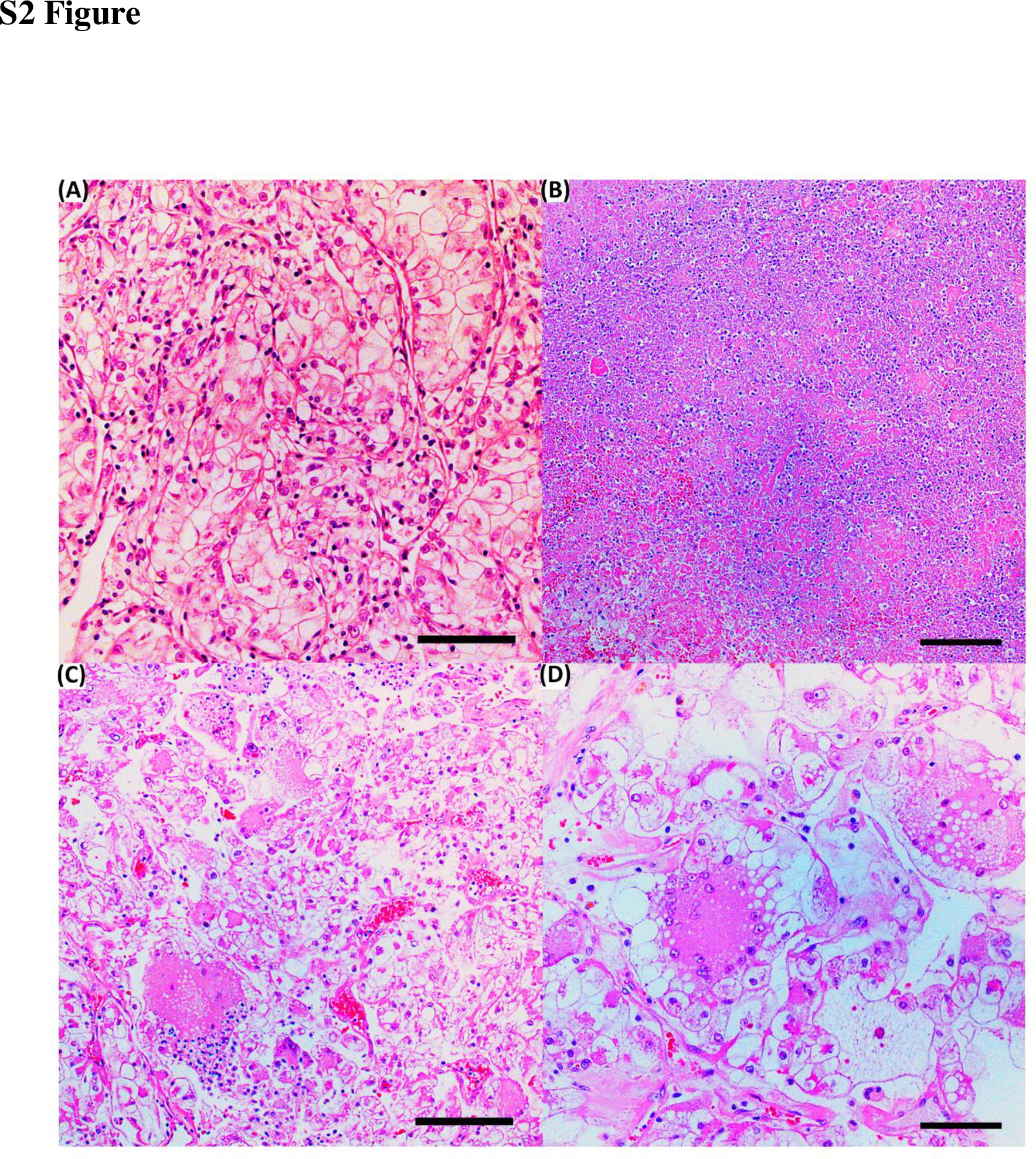

Supplement: S2 Fig — (A) The basic histopathological pattern of the cancer case was clear cell renal cell carcinoma. (B) In about 60% of the tumor tissue extensive areas of necrosis could be seen. (C and D) Areas displaying very unusual features where the cancer cells had coalesced into syncytial, multinuclear and discohesive cancer cells with fusion of the lipid laded cytoplasms. All images were stained by Hematoxylin/Eosin, Scale bars in A-C: 100 μm, in D: 200 μm. (TIF) [file pone.0279578.s002.tif]

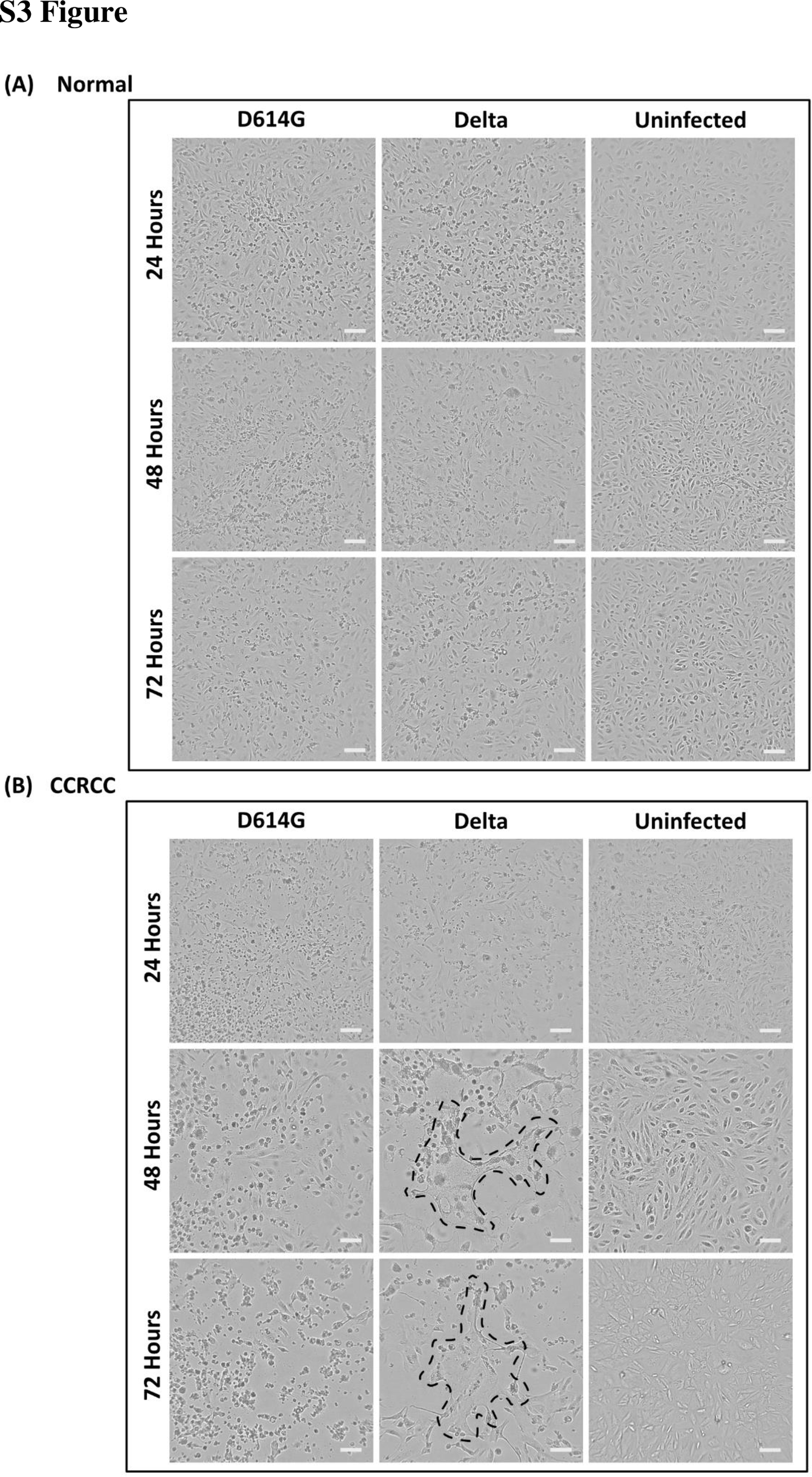

Supplement: S3 Fig — Representative images of (A) normal tubular epithelial cells and (B) clear cell renal cell carcinoma cells after exposure to the SARS CoV-2 variants D614G or delta. Uninfected is mock control. After 48 hours of exposure to the delta variant a distinct formation of syncytia is seen in the CCRCC cultures. Hatched lines mark syncytial cancer cells. Scale bar = 200μm. (TIF) [file pone.0279578.s003.tif]

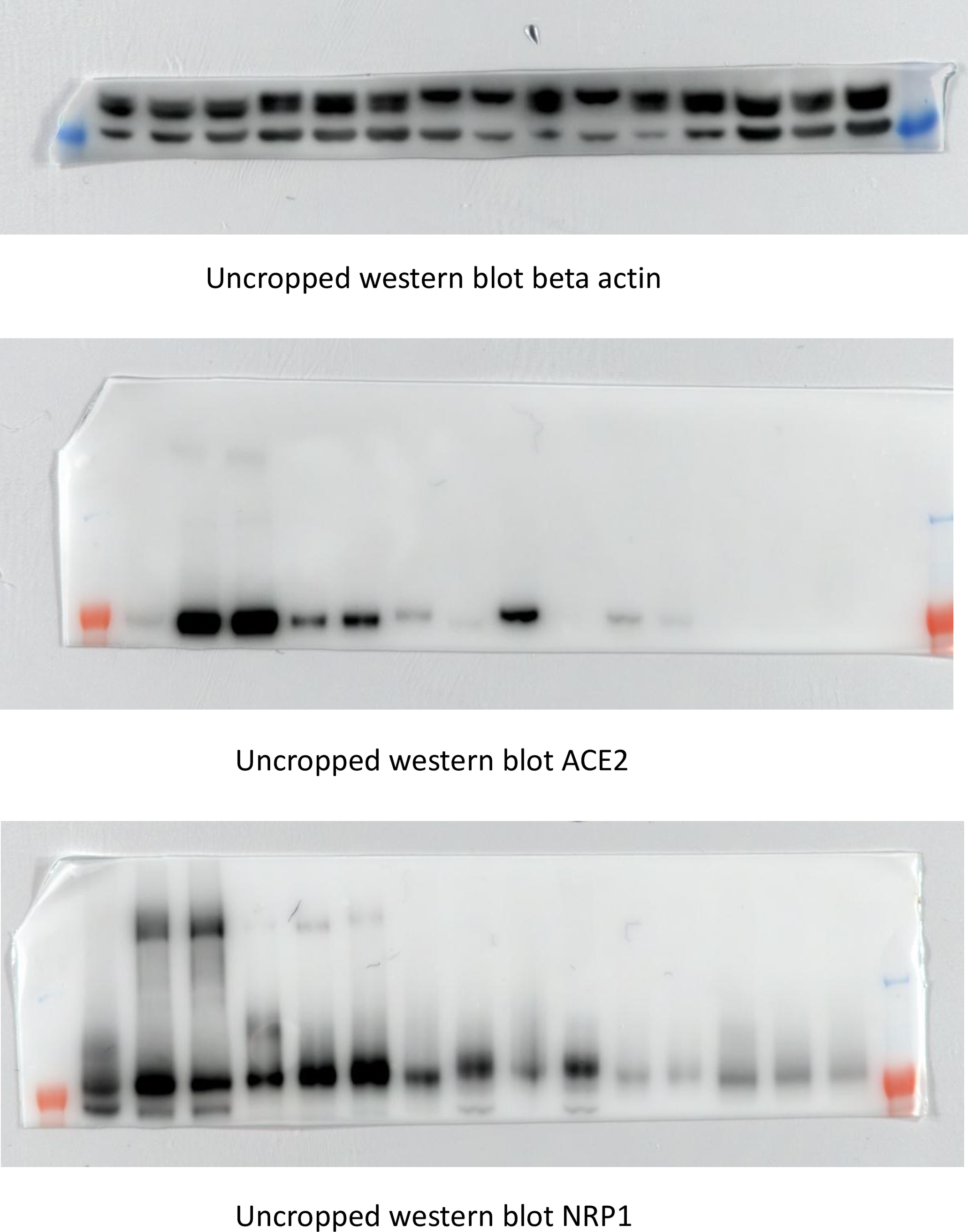

Supplement: S1 Raw images — (ZIP) [file pone.0279578.s004.zip › Fig8.tif]

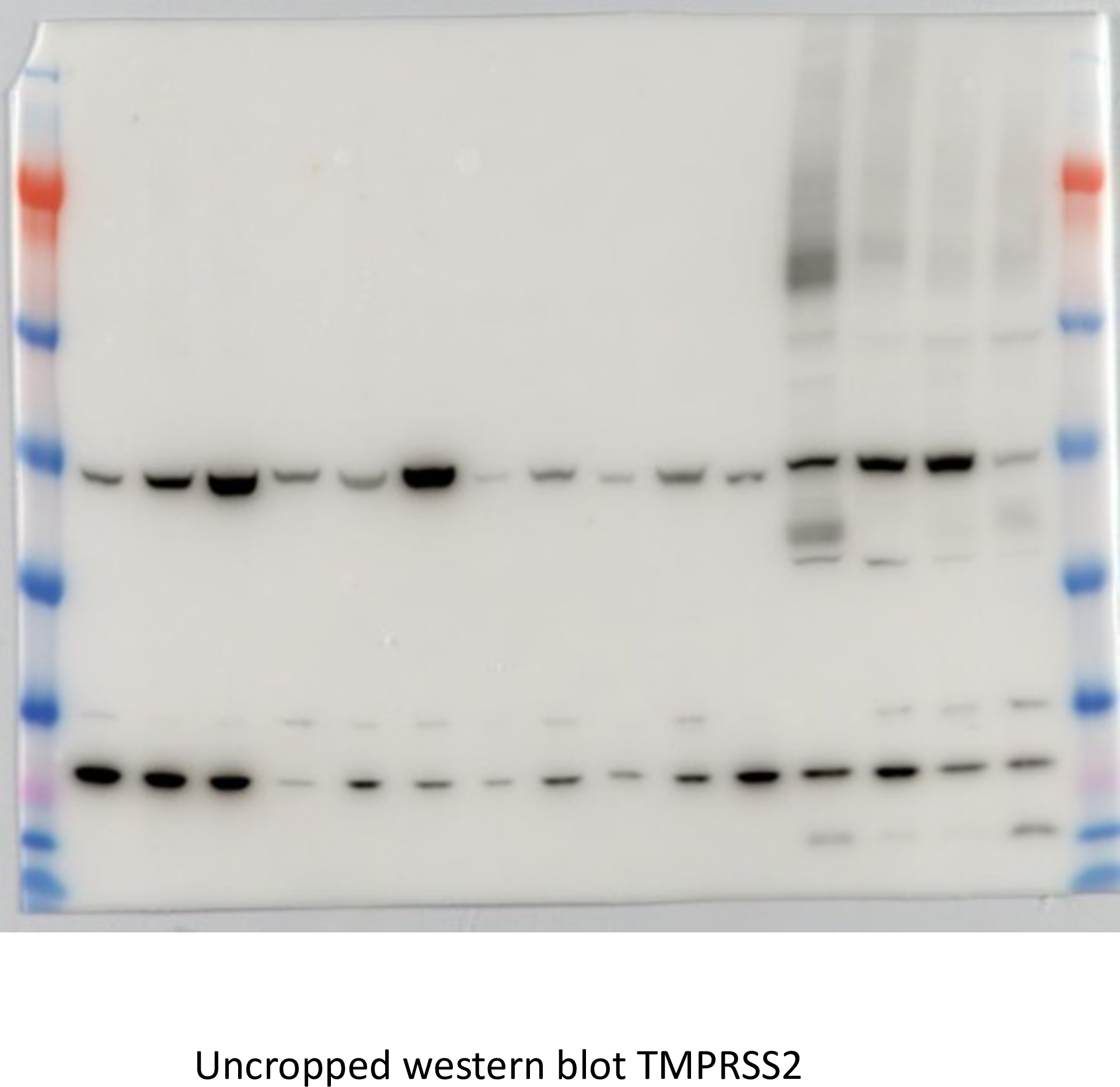

Supplement: S1 Raw images — (ZIP) [file pone.0279578.s004.zip › Fig9.tif]
