# Supplementary material for: SARS-CoV-2 replicates and displays oncolytic properties in clear cell and papillary renal cell carcinoma
Source: PLoS One. 2023 Jan 3;18(1):e0279578. doi: 10.1371/journal.pone.0279578 (PMC9810192; doi:10.1371/journal.pone.0279578)
Supplement: S1 Table — (DOCX) [file pone.0279578.s005.docx]

**S1 Table: Clinical characteristics of the patients included in the study**

| Case | Sex | Age (years) | Diagnosis | ISUP-grade | pTNM |
| --- | --- | --- | --- | --- | --- |
| CCRCC177 | Male | 61 | CCRCC | 1 | pT1a |
| CCRCC716 | Female | 70 | CCRCC | 2 | pT3 |
| CCRCC863 | Female | 58 | CCRCC | 2 | pT1b |
| CCRCC960 | Male | 60 | CCRCC | 2 | pT1a |
| PRCC545 | Male | 31 | PRCC type 2 | 2 | pT1b |
| PRCC769 | Male | 75 | PRCC type 1 | 2 | pT1a |
| PRCC993 | Male | 31 | PRCC type 1 | 2 | pT1a |
| PRCC754 | Male | 82 | PRCC type 2 | 2 | pT1b |
| CHRCC227 | Female | 41 | CHRCC | - | pT1b |
| CHRCC992 | Male | 69 | CHRCC | - | pT1a |
| CHRCC708 | Male | 59 | CHRCC | - | pT2 |
| CHRCC1033 | Male | 86 | CHRCC | - | pT3a |

CCRCC = clear cell renal cell carcinoma, PRCC = papillary renal cell carcinoma and CHRCC = chromophobe renal cell carcinoma
