# Supplementary material for: SARS-CoV-2 replicates and displays oncolytic properties in clear cell and papillary renal cell carcinoma
Source: PLoS One. 2023 Jan 3;18(1):e0279578. doi: 10.1371/journal.pone.0279578 (PMC9810192; doi:10.1371/journal.pone.0279578)
Supplement: Attachment — Submitted filename: Response to reviewers D 22 06970 R1.pdf [file pone.0279578.s007.pdf]

UNIVERSITY OF GOTHENBURG
THE SAHLGRENSKA ACADEMY

December 7th, 2022

Birke Bartosch
Academic Editor,
PLoS ONE

Dear Academic Editor Bartosch,

Thank you for the editorial handling and further reviews of our manuscript entitled:
SARS-CoV-2 replicates and displays oncolytic properties in clear cell and papillary renal cell carcinoma (PONE-D-22-06970R1) by Oi Kuan Choong et al.

We are very grateful for the constructive comments and criticisms. Below we address the comments by the editor and the further suggestions and concerns by reviewer #1. We interpret reviewer #2 as being satisfied with the current version. We believe that it has resulted in a further improvement of the manuscript. We have also edited the manuscript and figures in accordance with the journal guidelines, to the best of our ability. We hope that you and the reviewers find the measures taken adequate and that the current form of the manuscript is acceptable for publication.

Response to comments by the academic Editor

1. As suggested by reviewer 1, moving the CHRCC IHC from the supplementary into Figure 1 would make a good comparison of receptor expressing versus low/no expressing cells.
Response: We agree that this would indeed simplify the comparison of the expression of receptors and accessory factors. We have therefore moved the CHRCC staining from the supplemental section into figure 2D and changed the figure legends accordingly.

2. Furthermore, if PCR data on CHRCC are available, it would be useful to include them into Fig 3A.
Response: In figure 3A we show that primary cells from clear cell and papillary RCC propagate SARS-CoV-2 virus. We absolutely agree that a comparison with primary cultures of chromophobe RCC (CHRCC) would be of clear interest. CHRCC cannot be reliably cultured however, despite intense efforts from our side and to our knowledge few if any labs manage to establish primary cell cultures of CHRCC. Furthermore cell lines from CHRCC are not available. Therefore, we have no data from CHRCC cell cultures unfortunately.

Response to comments from Reviewer #1.
1. Figure 2: This figure includes IHC images of normal, CCRCC and PRCC cells but not CHRCC. It is more logical that S1A figure of CHRCC becomes part of Fig.2.
Response: We agree that it is more logical to have all the RCC stainings in one figure and have therefore moved the staining results for CHRCC from the supplemental figures to figure 2 D. We have also added H/E images of the same magnification as for figure 2A-C in order to achieve the same set up as for CCRCC and PRCC.

2. Figure 3A. All other cell types but CHRCC have been analyzed by PCR but CHRCC. If the authors have the data, it should be included (even if no infection is observed which could be expected).

Response: Regrettably, we could not show data from primary cultures of CHRCC. It would definitively have been appropriate as comparison. Primary CHRCC cells are difficult/ impossible to culture however, and we have not succeeded in establishing primary cultures of CHRCC cells. Also, there are no well characterized cell lines available to our knowledge. Therefore, we have no PCR data from the SARS-CoV-2 uptake and propagation experiment.

3. I still have a problem with the case study of 1 patient. Lines 335-336: "We can only suggest that SARS-CoV-2 may be causally connected to the unusual morphology". This is a pure speculation. Was this patient treatment naïve prior nephrectomy? Nothing can be concluded from this "unusual morphology" of 1 patient. However, this oriented the discussion toward viral oncolytic therapy.

Response: One case is definitely not enough to infer that SARS-CoV-2 causes RCC cell necrosis. We sought to modify our wording in the previous version, and we now try to soften our language further. First, we have now omitted the sentence of above completely (*"We can only suggest that SARS-CoV-2 may be causally connected to the unusual morphology"*

We also omit the sentence beginning at line 339, since we feel that is also a bit too bold:

"The goal of precision medicine is to selectively target cancer cells. For well over a century, oncolytic viral therapy has been researched."

We also add a new sentence to the discussion at line 346 to underscore that we are not implying a causal relationship. It reads as follows:

The isolated case described above does not prove causality for SARS-CoV-2 induced effects on CCRCC in patients.

If the reviewer still expresses concerns, we are more than happy to completely omit the case from the article. The reason for not doing so before re-submission this time is because the finding of multinucleated cells in the cancer tissue led us to make an additional to find out whether the virus may cause syncytialization of the cells. Perhaps omission would render the assay hanging in the air so to say. Second, we wish to alert the readership to keep an eye on possible unusual histological patterns in this patient category. Again, we are more than happy to erase the case completely if its presence hinders the appreciation of the paper. Not least since the central theme of the article focuses on SARS-CoV-2 tropism in RCC and we do not want that this is obscured.

Summing up, we hope that our responses to the comments and suggestions from the reviewers are regarded as adequate and that they have resulted in an improved manuscript.

Sincerely yours,

Martin Johansson

Martin Johansson
Professor of Pathology & Senior Consultant.
University of Gothenburg
Department of Clinical Pathology,
Sahlgrenska University Hospital
Gula Stråket 8, SE-41345 Göteborg, SWEDEN
mobile phone: +46-(0)73-6213802
email: martin.e.johansson@gu.se
