# Supplementary material for: SARS-CoV-2 replicates and displays oncolytic properties in clear cell and papillary renal cell carcinoma
Source: PLoS One. 2023 Jan 3;18(1):e0279578. doi: 10.1371/journal.pone.0279578 (PMC9810192; doi:10.1371/journal.pone.0279578)
Supplement: Attachment — Submitted filename: Response to Reviewers PONE D 22 06970 version TC.docx [file pone.0279578.s006.docx]

| Birke Bartosch  Academic Editor,  **PLoS ONE** | Martin Johansson Professor of Pathology, **University of Gothenburg**  [martin.e.johansson@gu.se](mailto:martin.e.johansson@gu.se)  Phone: +46(0)736213802  16^th^ of September 2022 |
| --- | --- |

**Rebuttal letter for the manuscript: *SARS-CoV-2 replicates and displays oncolytic properties in clear cell and papillary renal cell carcinoma* (PONE-D-22-06970)**

Dear Academic Editor Bartosch,

Below we present the responses to each of the points raised by the editor and the reviewers. The actions and responses are detailed in a point-by-point list below. We have also to the best of our ability edited the manuscript and figures in accordance with the journal guidelines.

EDITORIAL COMMENTS.

When submitting your revision, we need you to address these additional requirements.

*1. Please ensure that your manuscript meets PLOS ONE's style requirements, including those for file naming. The PLOS ONE style templates can be found at*

*https://journals.plos.org/plosone/s/file?id=wjVg/PLOSOne_formatting_sample_main_body.pdf and*

[*https://journals.plos.org/plosone/s/file?id=ba62/PLOSOne_formatting_sample_title_authors_affiliations.pdf*](https://journals.plos.org/plosone/s/file?id=ba62/PLOSOne_formatting_sample_title_authors_affiliations.pdf)*".*

**Response:** We have ensured that the manuscript meets the style requirements described above.

*2. We note that the grant information you provided in the ‘Funding Information’ and ‘Financial Disclosure’ sections do not match. When you resubmit, please ensure that you provide the correct grant numbers for the awards you received for your study in the ‘Funding Information’ section.*

**Response:** We apologize for the irregularities in format regarding funding and have now added complete funding information to the appropriate section. The acknowledgements section has therefore been removed.

*3. Thank you for stating the following in the Acknowledgments Section of your manuscript: “This study was supported by grants from the Swedish Cancer Society (Cancerfonden), the National Association against Kidney Diseases, Swedish Government Funding of Clinical Research within the National Health Service (ALF), the strategic research program BioCARE, the Research Fund (R&D) at Skaraborg Hospital, Skövde, Sweden and the Healthcare Committee, Region Västra Götaland, Sweden.” We note that you have provided funding information that is not currently declared in your Funding Statement. However, funding information should not appear in the Acknowledgments section or other areas of your manuscript. We will only publish funding information present in the Funding Statement section of the online submission form. Please remove any funding-related text from the manuscript and let us know how you would like to update your Funding Statement. Currently, your Funding Statement reads as follows:*

*“RJ: the Healthcare Committee, Region Västra Götaland, Sweden - 273289.*

*The funders had no role in study design, data collection and analysis, decision to publish, or preparation of the manuscript.”*

*Please include your amended statements within your cover letter; we will change the online submission form on your behalf.*

**Response:** All funding related text has been removed from the acknowledgements section. Instead, this information has been added to the appropriate section in the submission form (see point 2 above).

*4. We note that you have stated that you will provide repository information for your data at acceptance. Should your manuscript be accepted for publication, we will hold it until you provide the relevant accession numbers or DOIs necessary to access your data. If you wish to make changes to your Data Availability statement, please describe these changes in your cover letter and we will update your Data Availability statement to reflect the information you provide.*

**Response:** All data is provided in the supplemental section. Therefore, we need not use the repository information function. Thus, we ask if you could delete our previous statement in order to update our Data Availability statement.

*5. PLOS ONE now requires that authors provide the original ucropped and unadjusted images underlying all blot or gel results reported in a submission’s figures or Supporting Information files. This policy and the journal’s other requirements for blot/gel reporting and figure preparation are described in detail at https://journals.plos.org/plosone/s/figures#loc-blot-and-gel-reporting-requirements and https://journals.plos.org/plosone/s/figures#loc-preparing-figures-from-image-files. When you submit your revised manuscript, please ensure that your figures adhere fully to these guidelines and provide the original underlying images for all blot or gel data reported in your submission. See the following link for instructions on providing the original image data: https://journals.plos.org/plosone/s/figures#loc-original-images-for-blots-and-gels. In your cover letter, please note whether your blot/gel image data are in Supporting Information or posted at a public data repository, provide the repository URL if relevant, and provide specific details as to which raw blot/gel images, if any, are not available. Email us at plosone@plos.org if you have any questions.*

**Response:** The uncropped and unadjusted figures have been made available in the supporting information section of the paper.

REVIEWER No 1.

**Comments to the Author**

1. Is the manuscript technically sound, and do the data support the conclusions?

The manuscript must describe a technically sound piece of scientific research with data that supports the conclusions. Experiments must have been conducted rigorously, with appropriate controls, replication, and sample sizes. The conclusions must be drawn appropriately based on the data presented.

Reviewer #1: No

Reviewer #2: Yes

2. Has the statistical analysis been performed appropriately and rigorously?

Reviewer #1: N/A

Reviewer #2: Yes

3. Have the authors made all data underlying the findings in their manuscript fully available?

The [PLOS Data policy](http://www.plosone.org/static/policies.action#sharing) requires authors to make all data underlying the findings described in their manuscript fully available without restriction, with rare exception (please refer to the Data Availability Statement in the manuscript PDF file). The data should be provided as part of the manuscript or its supporting information, or deposited to a public repository. For example, in addition to summary statistics, the data points behind means, medians and variance measures should be available. If there are restrictions on publicly sharing data—e.g. participant privacy or use of data from a third party—those must be specified.

Reviewer #1: Yes

Reviewer #2: Yes

4. Is the manuscript presented in an intelligible fashion and written in standard English?

PLOS ONE does not copyedit accepted manuscripts, so the language in submitted articles must be clear, correct, and unambiguous. Any typographical or grammatical errors should be corrected at revision, so please note any specific errors here.

Reviewer #1: Yes

Reviewer #2: Yes

**Response to the reviewer comments to the author**

**Reviewer #1:** The study presented by Choong et al. implies that SARS-CoV-2 may replicate in renal cell carcinoma cells (CCRCC and PRCC) because these they express virus entry factors. Infection with SARS-Cov-2 resulted in cytopathic effect – syncytia formation. The manuscript is well written but I am afraid that some of the data presented suffer flaws and at some points there is overinterpretation of the results.

Here are my comments concerning the study:

**1.** Line 71: the authors conclude that their data “provide insights into SARS-CoV-2 infectivity in renal cell carcinoma and define a potential viral entry mechanism that could be used to target renal carcinoma”. However, the latter is not shown experimentally nor sufficiently discussion in the Discussion section. Moreover, in the Discussion section, the authors widely stress on oncolytic viral therapy implicating SARS-CoV-2. Such notion is not supported experimentally. Do cancer cells strongly overexpress viral entry factors compared to normal cells? How will be the specific targeting of cancer cells achieved? Indeed, measles virus (the vaccine strain!) has been used in such therapies, but this is justified by the strong expression of MCP (CD46) in cancer cells and the non-pathogenicity of the virus which is not the case with SARS-CoV-2.

**Response:** We thank the reviewer for this comment. We agree that we have overstated the interpretation of our results and that a more measured approach serves the manuscript better. To this end we have rewritten line 70-71 (*Our data provide insights into SARS-CoV-2 infectivity in renal cell carcinoma and define a potential viral entry mechanism that could be used to target renal cell carcinoma.).*

The new wording in line 70-71 is: Our data provide insights into SARS-CoV-2 infectivity in renal cell carcinoma and that the virus causes a distinct cytopathogenic effect.

We have also rewritten relevant parts of the discussion regarding oncolytic therapy in order to temper the conclusions. Specifically, we deleted the sentence beginning on line 346: (*We suggest that the mechanism presented in this paper may be used in a similar fashion*) and line 347-48: (*Another possibility may be to use the receptor apparatus of a modified attenuated virus to deliver therapeutic nucleotides or a similar payload to the cancer cells*).

The new text is: “Whether SARS-CoV-2 uptake and propagation in RCC cells could elicit a similar response is uncertain. Usage of this uptake mechanism is questionable due to the the pathogenicitity of the virus and lack of selectivity due to expression in normal organs.” Line 356-359.

Based on bioinformatic analysis of the TCGA data set we do believe that RCC is unusual in its expression of the entry factors for SARS-CoV-2. This we can corroborate using tissue micro arrays of a large number of RCC cases. This in turn reflects the native expression of these factors in the proximal tubular epithelium of the kidney, a colocalization not shared by many organs. There are however other tissues expressing these factors, so it is not entirely specific and selective for delivery to RCC cells. The overly speculative concluding sentence of the discussion section has therefore also been deleted, line 350-351: (*We suggest that the uptake mechanism for SARS-CoV-2 virus may be exploited to mediate uptake of therapeutic compounds or nucleic acids into renal cell carcinoma)* and changed to: “The cytopathogenic effect causes RCC cell lysis and the delta-variant of the virus causes syncytialization of the cancer cells.”, now line 363-364.

**2.** Concerning the Discussion, the authors are not the first to show that SARS-CoV-2 entry factors are found in renal cells. This are just some examples of other studies: PMID:32903321 (TMPRSS2), PMID:19736548, PMID:11566082 (NRP1), and for ACE-2 there are multiple papers.

**Response:** We thank the reviewer for this point, we are aware of this, but regret that we have failed to communicate this in the manuscript. We do mention in the introduction, that the factors are expressed at mRNA level in kidney tissue, but regarding protein expression we were less clear. We have added the references PMID 11566082 and PMID 32903321 to the reference list in order to more clearly acknowledge previous histological work. The sentence describing this (line 90-92) has been changed to account for this. New sentence in line 92-93.

**3.** Figure 1C: I am afraid I do not see the expression of TMPRSS2 in normal tissues on the blot as stated by the authors: line 226. The authors also stated that “NRP1 expression was robust across all cases” (Line 228). This is not the case for CHRCC and some of PRCC samples. A better blot must be provided, and also a quantification of the blot by densitometry.

**Response:** We agree that the western blot for TMPRSS2 is definitely not the easiest to interpret. To amend for this, we tested alternative antibodies for TMPRSS2 on the protein lysates. We chose anti-TMPRSS2 from Thermo Fisher (1:1000, MA5-35756). This resulted in a much better blot with improved signal and we have replaced the inferior blot with the new one in a new Figure 1C. Information regarding this has been added to the materials and methods section in the manuscript (line 167-168).

As suggested by the reviewer, we also performed densitometry of the bands in order to quantify ACE2, NRP1 and TMPRSS2, the results from this have also been added to figure 1C and to the figure legend (line 478-479). Finally, we have changed line 226-228 in the results section to reflect the improvements suggested by the reviewer. It now reads:

The ACE2 protein was clearly detectable by three out of four CCRCC and PRCC cases and was undetectable in CHRCC lysates. NRP1 expression was strongly expressed by three of four CCRCC and PRCC cases, and at lower intensity by chromophobe RCC. Samples from CCRCC, PRCC and CHRCC expressed TMPRSS2 protein. (now line 229-232)

**4.** Figure 2A: Use arrows to indicate different histological structures.

**Response:** This suggestion improves evaluation of the images. For clarity figure 2A has been added the letter G to indicate glomeruli, arrows for examples of proximal tubules and asterisks for collecting ducts. This has also been added to the figure legend at line 485-486.

**5.** Line 246: “We also formalin” The verb is missing…

**Response:** we have changed the sentence to the more clear-cut sentence: “Cells from the CCRCC derived cell line RCC4 were also fixed by formalin and paraffin embedded.” This is now the text at line 251-253.

**6.** Figure 3B: The data of syncytia formation in PRCC is not convincing (the microscopic image). Moreover, there is no correlation between the cell culture data (slightly visible syncytia) and the PCR data (strong expression) for PRCC.

**Response:** We are grateful for the opportunity to clarify the figures. Supplemental Figure 3B does not show syncytia in cultured PRCC cells, it shows the formation of syncytia in cultured primary CCRCC cells. We agree that the image could be improved and we have therefore hatched the perimeter of two distinct syncytialised CCRCC cells in supplemental figure 3B and description added to figure legend (line 525). Judging from figure 1C and figure 3A, the levels of attachment proteins and SARS-Cov-2 replication seem roughly similar between normal tubular epithelial cells and primary CCRCC cells. The protein expression is slightly lower in the CCRCC cells, but not significantly so.

**7.** I do not understand the including of the case study of 1 patient within this report. There is absolutely no proof that syncytia formation is due to SARS-CoV-2 (that the patient had 1 year before). Research for viral antigens was not performed. Moreover, syncytia formation is directly linked to cell lysis. This is even seen in the in vitro data on FigS3 B (almost no cells left).

**Response:** We agree with the concerns of the reviewer. We included the case, knowing that the number is too low to form a conclusion, especially since the active infection was a year previously. Having discovered that SARS-CoV-2 virus infects RCC cells in vitro, ideally, we would have liked to test the uptake in another system. The gold standard would be to infect a PdX mouse model with SARS-CoV-2. Since this is out of reach at this point, we reached out to the department of Urology asking if they had performed surgery on a patient with a proven SARS-CoV infection. They could only identifty one cases from their records. The histology being very unusual, we decided to add it to the paper in order to notify the pathology community about the possible morphology of a passing SARS-CoV-2 infection in RCC. The syncytia of the RCC-cases were definitely associated with cell lysis and outright necrosis of the RCC-tissue. This led us to design the experiment where we assessed if this could be repeated in vitro by a SARS-CoV-2.

As pointed out, we definitely understand the concerns of the reviewer and have added an additional sentence underscoring the speculative nature of the presented case at line 340-342 with the following wording:

We are aware of the anecdotal nature of this RCC case and can therefore not draw any conclusions regarding potential effects in human RCC.

We have also deleted the following sentence (line 331): *We suggest that this finding strengthens our assumption that SARS-CoV-2 might infect RCC also in patients*

**8.** In the discussion section (Lines 312-316) the authors say that “uptake and effect were not always clearly correlated to ACE-2 expression and that additional factors play role in causing cytopathogenic effect (CPE) in cell culture”. CPE is usually due to expression of viral receptors on uninfected cells and the expression of viral glycoproteins in infected cells. What is the expression of spike in these cells?

**Response:** This is a very interesting comment and suggestion. We would like to assess the expression of spike protein and accessory glycoproteins in the infected RCC and normal epithelial cells. However, we have not secured histological material or protein lysates from the infection assay, which precludes adding this data to the present study.

_____________________________________________________________________

**Reviewer #2:** The manuscript by Choong et al describes a very promising phenomenon: oncolytic activity of SARS-CoV-2 against renal carcinoma variants. The authors used bioinformatic analysis of existing RNAseq data and showed that both normal renal cells and cells of two renal carcinoma types expressed both ACE2 receptor and NRP1 co-factor required for SARS-CoV-2 entry. Then they verified these results by western blot analysis using normal and tumor renal cells. A correct (using a negative strand-specific RNA amplification) RT-qPCR analysis revealed that the same cell lines were permissive for virus, and in cases of high infection titers cytophathic effect is observed. The authors demonstrate formation of syncytia in renal tissue from a patient with previous COVID-19 infection, despite signs of the infection at a moment of analysis. In the discussion the authors speculate that SARS-CoV-2 can act as oncolutic viruses and may trigger tumor lysis in cancer patients with COVID-19, as observed for colon cancer patients. The manuscript is carefully and clearly written. All conclusions are based on presented experimental data. The topic definitely merits publication. Other groups have shown that SARS-CoV-2 does not always display cytophatic effect (i.e., in case of hepatocellular carcinoma Huh7 cell line).
I would suggest publishing the study after answering two short questions:

**1.** Was cytophatic effect quantified Ii.e. by MTT assay, staining with annexin V or propidium iodide)?

**Response:** This is a very good point. We used the method preferred by the virologists at the virology department of the university hospital. Therefore, we assessed the cells by inspection and photography. The cytopathic effect was pronounced and easy to see and compared to infection controls and uninfected cells. Virus replication in cell culture as determined by visual cytopathic effect was further confirmed by RT-PCR. We did not run the suggested assays, partially due to infectivity issues.

**2.** Can the authors speculate about the mechanisms that control cytopathic effect of the virus? or in authors opinion the effect is merely due to high replication rate? In our personal experience some cells lines survive during the infection, despite high replication rates.

**Response:** For SARS-CoV-2 exposed kidney derived cell lines we observe a clear correlation between a high replication rate and CPE. For other cell lines such as calu3 more virus is necessary to induce CPE and a high viral replication rate.

A sentence regarding this has been added to the discussion section starting at line 330 with the following wording:

The effect on the primary RCC cells was similar in magnitude to that of the kidney derived cell lines used to propagate SARS-CoV-2 virus in the clinical setting at the department of virology. Other cell lines used, such as calu3 require higher viral load for CPE and high replication (322-325)*.*

In conclusion we hope that the changes and additions described above have met the expectations of the editor and the reviewers. We wish to thank all involved for their work in the constructive review of this manuscript.

Best Regards

Martin Johansson

Professor of Clinical Pathology

Institute of Biomedicine/Dept of Clinical Pathology

Postal Address: SU Sahlgrenska, 41345 Göteborg
Visiting Address: Gula stråket 8 SU/Sahlgrenska ,

41345 Göteborg Sweden

Cell phone: +46-(0)736-213802

email: martin.e.johansson@gu.se
